# New biomarkers of Kawasaki disease identified by urine proteomic analysis

**DOI:** 10.1002/2211-5463.12563

**Published:** 2018-12-20

**Authors:** Hui‐Min Hu, Hong‐Wu Du, Jia‐Wen Cui, De‐Qin Feng, Zhong‐Dong Du

**Affiliations:** ^1^ Department of Cardiology Beijing Children's Hospital Capital Medical University Beijing China; ^2^ Department of Pediatrics Beijing Tongren Hospital Capital Medical University Beijing China; ^3^ School of Chemistry and Biological Engineering University of Science & Technology Beijing China; ^4^ Institute of Microbiology Chinese Academy Science Beijing China; ^5^ Shunyi Maternal and Children's Hospital of Beijing Children's Hospital China

**Keywords:** biomarker, Kawasaki disease, pneumonia, protein, urine proteomics

## Abstract

Kawasaki disease (KD) is an acute systemic vasculitis that mainly afflicts infants and young children. The symptoms of KD are similar to those of various febrile diseases. Here, we attempted to develop accurate diagnostic biomarkers of KD by performing urine proteomic analysis of samples from healthy controls, patients with KD, and patients with another febrile disease, pneumonia (two patients). We identified differentially expressed proteins (DEPs) in KD as compared to normal controls. We also constructed functional annotation and protein–protein interaction (PPI) networks of DEPs in KD and pneumonia. DEPs common to both KD and pneumonia were identified, as well as DEPs specific to KD. Compared to normal control, 43 and 62 DEPs were identified in KD and pneumonia, respectively. Serine hydroxymethyltransferase 1 is a hub protein of the KD‐specific PPI network. Thirteen DEPs common to both KD and pneumonia and 30 DEPs specific to KD were identified. Of these, the expression of eight DEPs could cluster normal and pneumonia samples into one group and cluster KD samples into another group based on hierarchical clustering. Our study identified several DEPs that may play a role in KD and that may serve as diagnostic biomarkers to distinguish patients with KD from both normal control and other febrile diseases.

**Abbreviations**
CAA: coronary artery abnormality
DEP: differentially expressed protein
FDR: false discovery rate
GO: gene ontology
IGHV3‐23: immunoglobulin heavy variable 3‐23
KD: Kawasaki disease
KEGG: Kyoto Encyclopedia of Genes and Genomes
PECAM‐1: platelet endothelial cell adhesion molecule 1
PGLYRP1: peptidoglycan recognition protein 1
PPI: protein–protein interaction
RPLC: reversed‐phase liquid chromatography
SHMT1: serine hydroxymethyltransferase 1

Kawasaki disease (KD) is an acute febrile mucocutaneous lymph node syndrome that is characterized by systemic febrile vasculitis symptoms and mainly occurs in infants or young children 1, 2. Approximately 15–20% of patients with KD who are not diagnosed in time or without treatment may develop coronary artery abnormalities (CAAs) 3. Moreover, KD is the most common cause of acquired heart disease in infants and young children 4.

Despite efforts over several decades, the etiology of KD remains elusive and there is no accurate diagnostic biomarker of KD. KD is solely diagnosed based on symptoms including fever for more than 5 days accompanied with manifestations such as skin rash, red eyes, red lips and mouth, swollen and red hands and feet, and swollen lymph nodes in the neck area 3. However, the manifestations of KD are similar to many other febrile diseases such as pneumonia, scarlet fever, viral infection, juvenile idiopathic arthritis and measles, which may lead to misdiagnosis or delayed diagnosis of KD, especially, the incomplete KD 5. Hence, it is essential to develop accurate diagnostic biomarkers of KD that could distinguish KD from not only normal controls but also other febrile diseases with similar symptoms.

Pneumonia has typical symptoms, signs and imaging changes, and there are no swollen lymph nodes in the neck area and rash. Therefore, pneumonia is generally not confused with incomplete KD. Proteomic analysis is an emerging and promising field of research. Proteomic analyses could be used to explore the pathogenesis of various diseases and make a contribution to developing potential diagnostic biomarkers and therapeutic targets. Urine is a source of potential markers of disease and urine proteomic analysis has been used to identify key proteins in various diseases such as kidney disease 6, thrombosis 7 and acute coronary syndromes 8. A few studies have explored pathogenesis by using proteomic analysis, and filamin C and meprin A were reported to be potential biomarkers of KD 9, 10.

To further research more accurate and sensitive diagnostic markers of KD, we analyzed the urine proteomes of patients with KD, normal control and patients with another febrile disease, pneumonia. Our study may provide new clues for understanding the mechanism and developing new diagnostic and therapeutic strategies of KD.

## Methods

### Patients

Four patients with KD (KD 1–4), two patients with pneumonia (Pneumonia 1 and 2) and two normal controls (normal 1 and 2) were enrolled in this study. The diagnostic criteria of KD are fever for at least 5 days combined with at least four of the five following symptoms: (a) bilateral conjunctival injection; (b) erythema of the oral mucosa and/or lips; (c) rash; (d) swelling and redness of the hands and/or feet; (e) an enlarged cervical lymph node of at least 1.5 cm. In addition, patients with fever, three of the five above symptoms, and coronary artery abnormalities according to echocardiogram or angiography could be diagnosed as having KD as well. Patients with fever and fewer than four of the five KD clinical symptoms were diagnosed as atypical or incomplete KD. Patient KD 1 was a 5‐year‐old boy who was diagnosed as KD combined with hydropericardium based on symptoms including fever for 6 days, swollen lymph nodes in the neck area, and red eyes; patient KD 2 was a 4‐year‐old boy who was diagnosed as KD combined with myocardial damage based on symptoms including fever for 6 days, swollen lymph nodes in the neck area, red eyes, rash, bayberry tongue, red lip, chap; patient KD 3 was a 3‐year‐old boy who was diagnosed as KD combined with injury of left coronary artery based on symptoms including fever for 5 days, swollen lymph nodes in the neck area, red eyes, rash; patient KD 4 was a 6‐year‐old boy who was diagnosed as incomplete KD combined with dilatation of left coronary artery based on symptoms including intermittent fever for 12 days and rash. Two patients with febrile pneumonia had symptoms of fever, namely a 3‐year‐old girl and a 4‐year‐old boy. Two normal controls were a 4‐year‐old boy and a 5‐year‐old girl. The informed consents of all participants were signed by their guardians. The research protocol was approved by the ethics committee of Beijing Children's Hospital, Capital Medical University. This research complied with the principles of the Declaration of Helsinki.

### Urinary protein extraction

Morning urine samples were collected from all the eight participants. All the urine samples were centrifuged at 5000 ***g*** for 30 min and the precipitates removed. The supernatants were precipitated at −4 °C overnight with three times volume cold acetone added. The precipitates were collected after 10 000 ***g*** centrifugation for 30 min and resuspended in lysis buffer (25 mm TEAB, 8 m urea, 2% Triton X‐100, 0.1% SDS, proteinase inhibitor) and centrifuged at 18 800 ***g*** for 1 h at 15 °C. The concentration of proteins in each urine sample was quantified by using 2‐D Quant Kit (GE healthcare, Princeton, NJ, USA).

### Protein digestion and iTRAQ labeling

Protein digestion and iTRAQ labeling were performed by using an ITRAQ reagent‐8plex multiplex kit (AB Sciex, Foster City, CA, USA) according to the manufacturer's instructions. Firstly, 100 μg protein was incubated at 37 °C in a water bath for 1 h with 4 μL reducing reagent added and then incubated at 37 °C in a water bath in the dark for an additional 30 min with 2 μL cysteine‐blocking reagent added. Secondly, the reaction mixture was transferred to a 10K centrifugal filter tube and centrifuged at 10 000 ***g*** for 20 min and the filtrate then collected, and the filtration was repeated three times. After 100 μL dissolution buffer was added, the solution was centrifuged at 10 000 ***g*** for 20 min and then the solution in the bottom of the filter tube was removed. This was repeated 3 times. Thirdly, 6 μg trypsin (Roche, Basel, Switzerland; dissolved with 50 μL dissolution buffer that was diluted 5‐fold) was added and the reaction mixture was incubated at 37 °C in a water bath for 15 h. Fourthly, the digested peptides were collected as a filtrate. According to the manufacturer's protocol, the KD, pneumonia and normal samples were labeled with 113–121 ITRAQ reagents. Finally, the pooled samples were analyzed by two‐dimensional liquid chromatography–tandem mass spectrometry.

### High‐pH reversed‐phase liquid chromatography separation

By using a high‐pH reversed‐phase liquid chromatography (RPLC) column (Gemini 5u C18 110Å, 250 × 4.6 mm, Phenomenex), the pooled mixture of iTRAQ labeled samples was fractionated. Samples of 120 μL were loaded onto the column in buffer A1 (ammonium formate, 2 mm, pH 10) six times. A 60 min gradient was performed with 2–95% buffer B1 (ammonium formate, 1.5 mm, pH 10, 80% HPLC grade acetonitrile) at a flow rate of 0.5 mL·min^−1^. A total of 60 fractions were collected at 1 min per fraction. The dried 60 fractions were re‐suspended by 20 μL 0.1% formic acid and pooled into 10 samples by combining fractions 1, 11, 21, 31, 41, 51; 2, 12, 22, 32, 42, 52; 3, 13, 23, 33, 43, 53; and so on. A total of 10 samples were centrifuged at 16 °C, at 16 000 ***g*** for 10 min. Three microliters of each sample was analyzed by nano liquid chromatography–tandem mass spectrometry (nano LC‐MS/MS).

### Nano LC‐MS/MS analysis

Each fraction was analyzed with an Easy‐spray column (Thermo Scientific, Wilmington, DE, USA; Easy‐nLC 1000) with parameters C18, 5 μm, 120 Å, 75 μm × 15 cm. Buffer A2 was 0.1% formic acid in water and buffer B2 was 0.1% formic acid in HPLC grade acetonitrile. The elution gradient was 3–90% buffer B2 at flow rate = 0.3 μL·min^−1^ for 40 min. An orbitrap fusion mass spectrometer (Thermo Scientific) was used to analyze eluted peptides from liquid chromatography. The MS data were acquired in the Orbitrap detector by using the top speed mode with the following parameters: Orbitrap resolution, 120 000 FWHM; cycle time, 3s; dynamic exclusion duration, 40 s; scan range, 350–1550 *m*/*z*.

### Differentially expressed protein analysis

After data processing, the differentially expressed proteins (DEPs) in both the KD and the pneumonia samples compared to normal control samples were identified with |Abundance Ratio ≥1.5 or <0.667. *P*‐value <0.05 was considered significant. Hierarchical clustering of the expression of DEPs was performed by using pheatmap in the r language (https://www.r-project.org/).

### Functional annotation of DEPs in KD and pneumonia

To further research the biological functions of the DEPs in KD and pneumonia (two patients) compared to normal control, functional annotation was performed. Gene Ontology (GO) and Kyoto Encyclopedia of Genes and Genomes (KEGG) pathway enrichment analyses were performed by using online GeneCoDis3 (http://genecodis.cnb.csic.es/analysis). False discovery rate (FDR) < 0.05 was set as the cut‐off of significance. Moreover, by using the String database (https://string-db.org/), a protein–protein interaction (PPI) network of DEPs in KD and pneumonia was obtained and visualized by cytoscape software (http://www.cytoscape.org/).

### Further research on the protein biomarkers in KD

Firstly, the common DEPs in both KD and pneumonia (two patients) compared to normal control were obtained. Then, hierarchical clustering of the expression profile of these common DEPs in KD, pneumonia and normal control was performed by using pheatmap in the R language.

Compared to normal control, KD‐specific DEPs in KD that did not belong to DEPs in pneumonia were obtained. Hierarchical clustering of the expression profile of these particular DEPs in KD, pneumonia (two patients) and normal control was performed by using pheatmap in the R language.

## Results

### DEPs in KD and pneumonia compared to normal control

**Figure 1:**
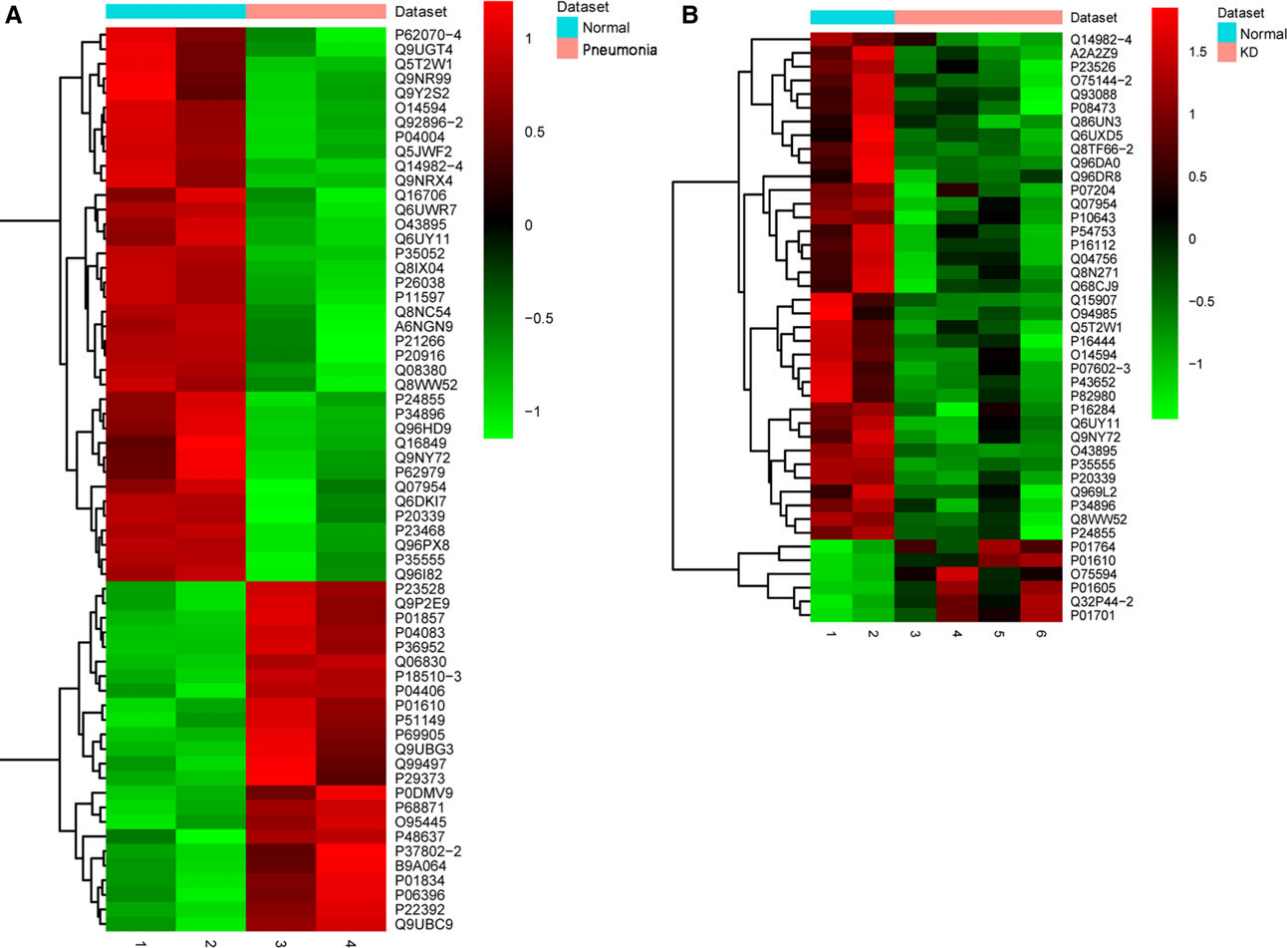
Hierarchical clustering analysis of the expression levels of DEPs in KD and pneumonia compared to normal control. Rows and columns represent the accession of each DEP and sample, respectively. The color scale indicates the expression level of each DEP. Red and green indicated up‐ and down‐regulation, respectively. (A) DEPs in pneumonia compared to normal control. (B) DEPs in KD compared to normal control.

A total of 1063 proteins could be quantified in KD and normal groups. A total of 1067 proteins could be quantified in pneumonia (two patients) and normal groups. Compared to normal control, 43 DEPs in KD (37 down‐regulated and six up‐regulated DEPs; Table 1) and 62 DEPs in pneumonia (38 down‐regulated and 24 up‐regulated DEPs; Table S1) were identified. Hierarchical clustering of DEPs in KD and pneumonia is displayed in Fig. 1.

**Table 1:** The differentially expressed proteins in KD compared to normal control. Abundance ratio is the ratio KD/normal control

Accession	Description	Abundance ratio	*P*
P35555	Fibrillin‐1, FBN1	−3.125	0.000
O43895	Xaa‐Pro aminopeptidase 2, XPNPEP2	−2.252	0.000
P20339	Ras‐related protein Rab‐5A, RAB5A	−2.890	0.003
Q8TF66‐2	Isoform 2 of leucine‐rich repeat‐containing protein 15, LRRC15	−2.463	0.005
Q96DA0	Zymogen granule protein 16 homolog B, ZG16B	−1.543	0.007
Q15907	Ras‐related protein Rab‐11B, RAB11B	−1.706	0.008
P43652	Afamin, AFM	−3.067	0.009
Q8WW52	Protein FAM151A, FAM151A	−1.835	0.010
A2A2Z9	Ankyrin repeat domain‐containing protein 18B, ANKRD18B	−2.415	0.010
Q07954	Prolow‐density lipoprotein receptor‐related protein 1, LRP1	−2.994	0.013
P82980	Retinol‐binding protein 5, RBP5	−2.933	0.014
O75144‐2	Isoform 2 of ICOS ligand, ICOSLG	−1.692	0.016
Q5T2W1	Na^+^/H^+^ exchange regulatory cofactor NHE‐RF3, PDZK1	−1.825	0.018
Q6UY11	Protein delta homolog 2, DLK2	−2.364	0.018
P16112	Aggrecan core protein, ACAN	−2.058	0.019
Q9NY72	Sodium channel subunit beta‐3, SCN3B	−2.262	0.019
O94985	Calsyntenin‐1, CLSTN1	−2.801	0.022
P24855	Deoxyribonuclease‐1, DNASE1	−1.577	0.022
O14594	Neurocan core protein, NCAN	−1.565	0.023
P23526	Adenosylhomocysteinase, AHCY	−2.283	0.023
P34896	Serine hydroxymethyltransferase, cytosolic, SHMT1	−2.193	0.024
P07602‐3	Isoform Sap‐mu‐9 of Prosaposin, PSAP	−4.000	0.028
P16444	Dipeptidase 1, DPEP1	−2.012	0.028
Q8N271	Prominin‐2, PROM2	−2.141	0.029
P10643	Complement component C7, C7	−2.037	0.029
Q32P44‐2	Isoform 2 of echinoderm microtubule‐associated protein‐like 3, EML3	4.808	0.032
P01764	Ig heavy chain V‐III region 23, IGHV3‐23	1.617	0.032
Q68CJ9	Cyclic AMP‐responsive element‐binding protein 3‐like protein 3, CREB3L3	−2.058	0.034
P01610	Ig kappa chain V‐I region WEA	2.913	0.035
Q93088	Betaine–homocysteine *S*‐methyltransferase 1, BHMT	−1.799	0.035
Q86UN3	Reticulon‐4 receptor‐like 2, RTN4RL2	−1.634	0.035
O75594	Peptidoglycan recognition protein 1, PGLYRP1	1.622	0.035
P54753	Ephrin type‐B receptor 3, EPHB3	−1.715	0.036
Q96DR8	Mucin‐like protein 1, MUCL1	−1.520	0.036
P01701	Ig lambda chain V‐I region NEW	3.769	0.037
P16284	Platelet endothelial cell adhesion molecule, PECAM1	−1.818	0.037
Q6UXD5	Seizure 6‐like protein 2, SEZ6L2	−2.392	0.038
Q14982‐4	Isoform 4 of opioid‐binding protein/cell adhesion molecule, OPCML	−1.600	0.038
P01605	Ig kappa chain V‐I region Lay	2.234	0.039
Q04756	Hepatocyte growth factor activator, HGFAC	−2.141	0.041
P08473	Neprilysin, MME	−1.883	0.043
Q969L2	Protein MAL2, MAL2	−1.754	0.045
P07204	Thrombomodulin, THBD	−1.582	0.050

### Functional annotation of DEPs in KD and pneumonia compared to normal control

**Figure 2:**
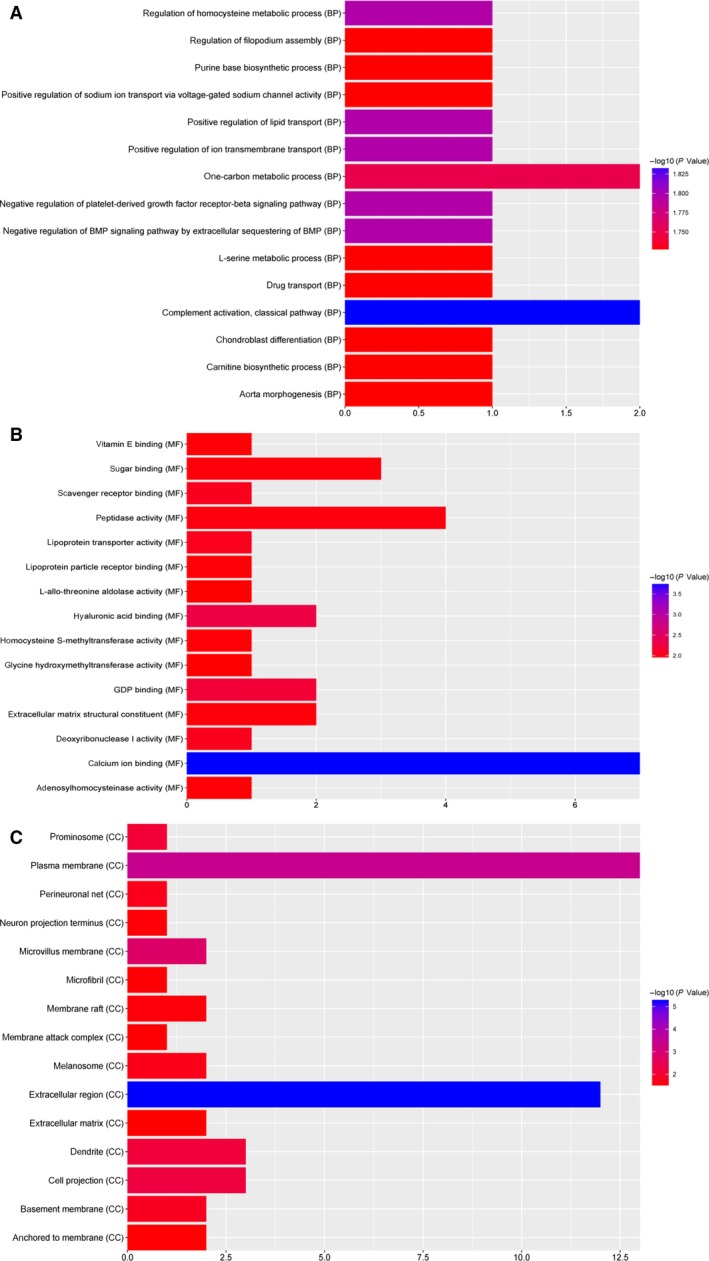
The top 15 most significantly enriched GO terms in DEPs of KD. The *x*‐axis represents the −log (*P*‐value) and the *y*‐axis represents the GO terms. (A) Biological process (BP); (B) molecular function (MF); (C) cellular component (CC).

Based on the GO enrichments, complement activation, classical pathway (FDR = 0.0146312), positive regulation of lipid transport (FDR = 0.0160379), extracellular region (FDR = 0.00000607909), calcium ion binding (FDR = 0.000190139) and hyaluronic acid binding (FDR = 0.000152742) were significantly enriched GO terms of the DEPs in KD (Fig. 2). Vasopressin‐regulated water reabsorption, cysteine and methionine metabolism (FDR = 0.000556988) and glycine, serine and threonine metabolism (FDR = 0.000439577) were the top three significantly enriched pathways of the DEPs in KD (Table 2). The renin–angiotensin system was a significantly enriched pathway in KD as well (Table 2).

**Table 2:** The significantly enriched pathways in KD compared to normal control

KEGG ID	Term	FDR	Accession (symbol)
KD			
04962	Vasopressin‐regulated water reabsorption	0.000	Q15907 (RAB11B), Q68CJ9 (CREB3L3), P20339 (RAB5A)
00270	Cysteine and methionine metabolism	0.004	Q93088 (BHMT), P23526 (AHCY)
00260	Glycine, serine and threonine metabolism	0.005	Q93088 (BHMT), P34896 (SHMT1)
04974	Protein digestion and absorption	0.014	O43895 (XPNPEP2), P08473 (MME)
00460	Cyanoamino acid metabolism	0.025	P34896 (SHMT1)
05010	Alzheimer's disease	0.040	Q07954 (LRP1), P08473 (MME)
00670	One carbon pool by folate	0.042	P34896 (SHMT1)
04614	Renin–angiotensin system	0.045	P08473 (MME)

Hydrogen peroxide catabolic process (FDR = 0.00000374185), lipoprotein metabolic process (FDR = 0.0000344958), extracellular region (FDR = 0.00000000556057), peroxidase activity (FDR = 0.00000459332) and haptoglobin binding (FDR = 0.000110832) were significantly enriched GO terms of DEPs in pneumonia (two patients).

### KD‐ and pneumonia‐specific PPI network

**Figure 3:**
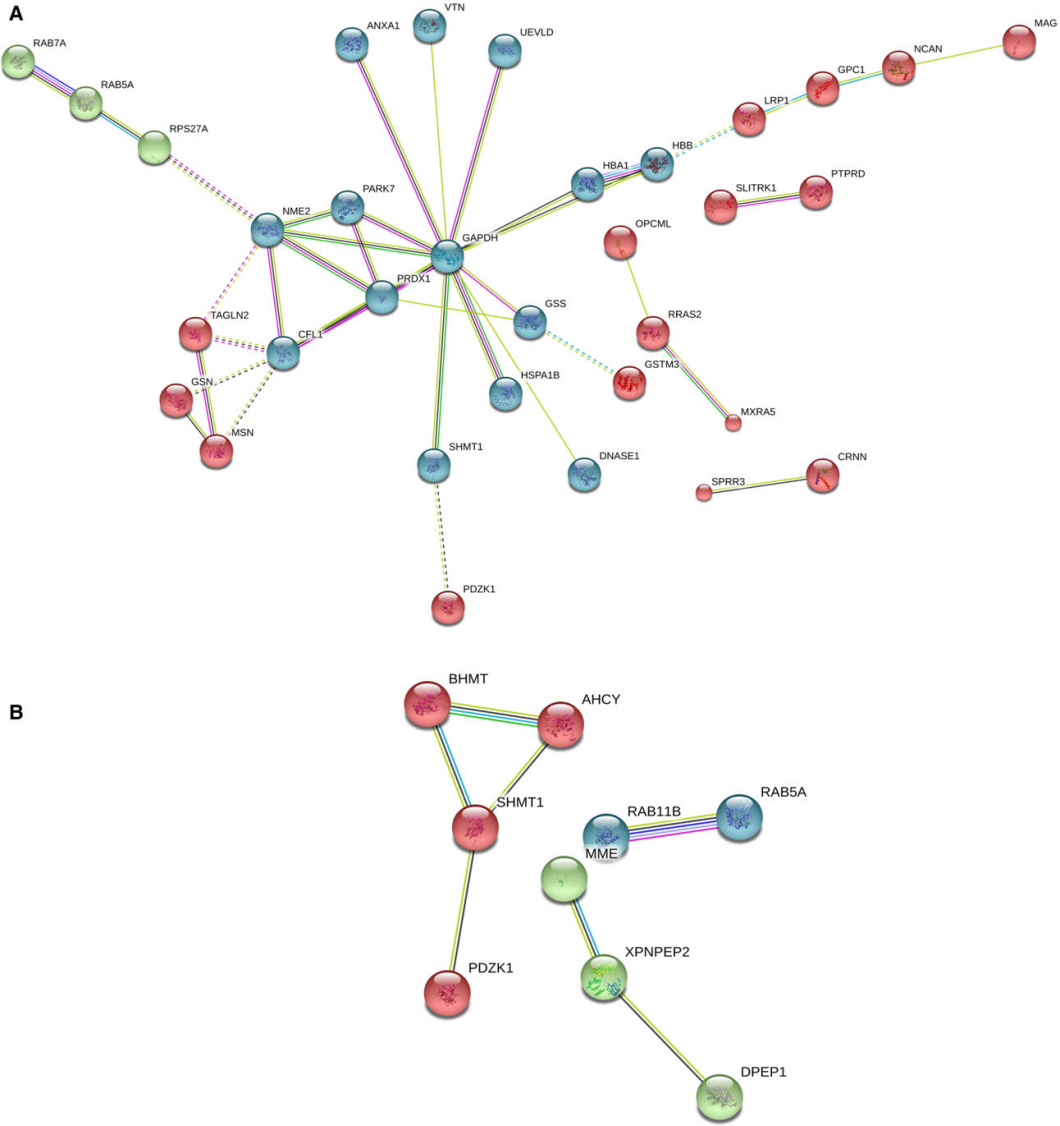
The KD‐ and pneumonia‐specific PPI network. (A) Pneumonia‐specific PPI network. (B) KD **‐**specific PPI network.

The pneumonia‐specific PPI network consisted of 33 nodes and 39 edges (Fig. 3). Glyceraldehyde 3‐phosphate dehydrogenase (GAPDH; degree = 13, closeness centrality = 0.54), Nucleoside diphosphate kinase B (NME2; degree = 6, closeness centrality = 0.44) and Cofilin‐1 (CFL1; degree = 6, closeness centrality = 0.42) were the three hub proteins of the pneumonia‐specific PPI network. The KD‐specific PPI network consisted of nine nodes and seven edges (Fig. 3). Serine hydroxymethyltransferase 1 (SHMT1; degree = 3, closeness centrality = 0.33), Xaa‐Pro aminopeptidase 2 (XPNPEP2; degree = 13, closeness centrality=0.54), Adenosylhomocysteinase (AHCY; degree = 2, closeness centrality = 1.0) and Betaine‐homocysteine S‐methyltransferase 1 (BHMT; degree = 2, closeness centrality = 1.0) were the four hub proteins of the KD‐specific PPI network.

### Common DEPs in both KD and pneumonia compared to normal control

**Figure 4:**
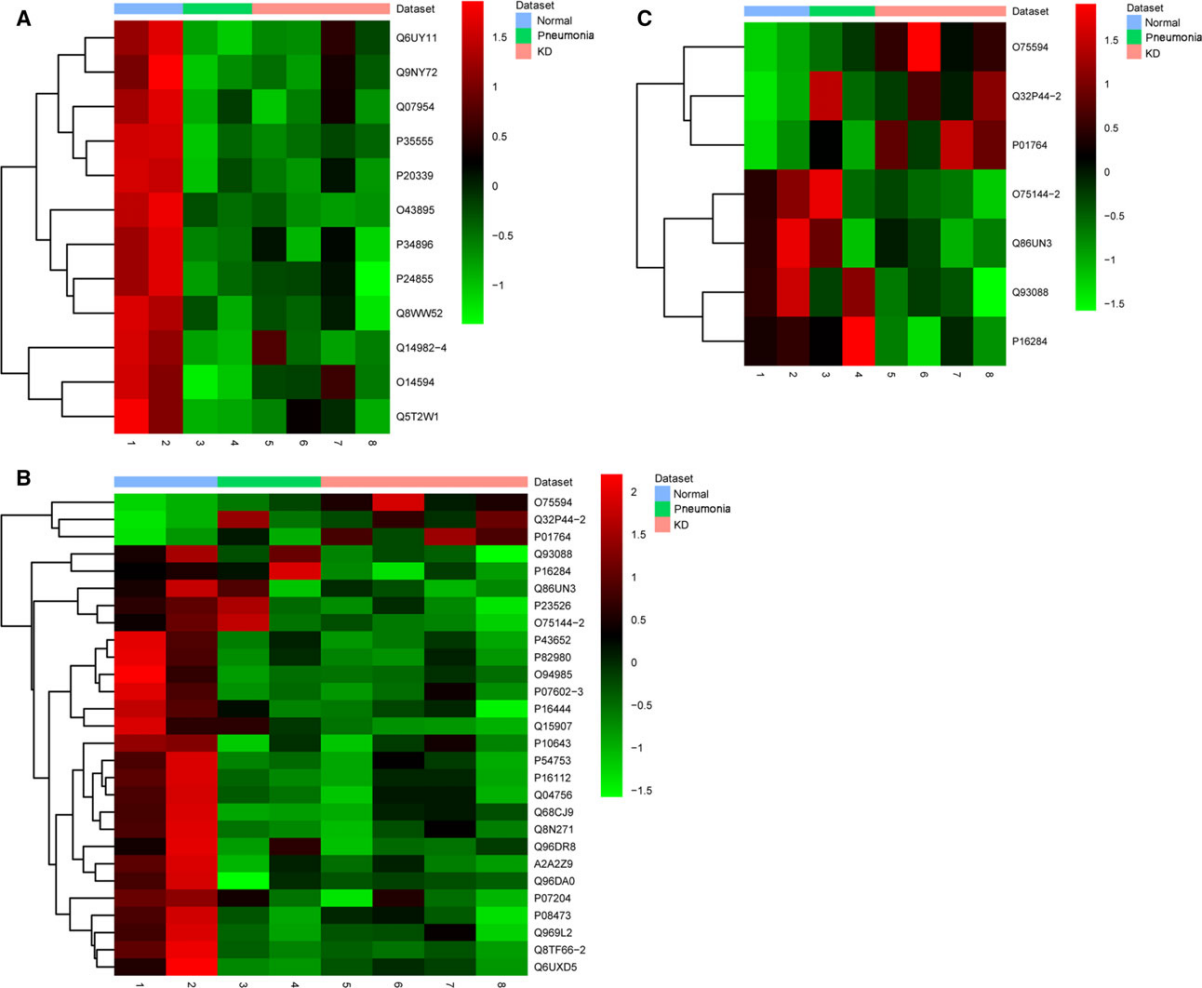
Hierarchical clustering analysis in KD, pneumonia and normal control. Rows and columns represented the accession of each DEP and sample, respectively. The color scale indicates the expression level of each DEP. Red and green indicate up‐ and down‐regulation, respectively. (A) Common DEPs in both KD and pneumonia compared to normal control; (B) KD‐specific DEPs that did not belong to DEPs in pneumonia; (C) eight candidate KD‐specific DEPs that did not belong to DEPs in pneumonia.

Compared to normal control, a total of 13 common DEPs in both KD and pneumonia (two patients) were obtained. One of these 13 DEPs had no symbol. Hierarchical clustering of the other 12 common DEPs in KD, pneumonia (two patients) and normal control is displayed in Fig. 4A. All these 12 DEPs were significantly down‐regulated in both KD and pneumonia (two patients) compared to normal control. The expression of these DEPs clustered the KD and pneumonia samples into one group and clustered the normal samples into another group.

### KD‐specific DEPs that did not belong to DEPs of pneumonia

A total of 30 common particular KD‐specific DEPs were obtained that did not belong to DEPs of pneumonia (two patients). Two of these 30 DEPs had no symbol. Hierarchical clustering of the other 28 particular DEPs in KD, pneumonia (two patients) and normal control is displayed in Fig. 4B. This hierarchical clustering showed that the expression of these 28 particular DEPs apparently clustered all samples into two groups. In these, KD and pneumonia samples were clustered into one group and normal samples were clustered the other group. This result indicated that the expression of these 28 DEPs could be used to distinguish normal groups and disease groups (KD and pneumonia groups (two patients)). Moreover, the expression of eight DEPs (PECAM1, PGLYRP1, AHCY, BHMT, EML3, ICOSLG, IGHV3‐23 and RTN4RL2) in normal and pneumonia groups (two patients) was quite different from that in the KD group (Fig. 4C). Hierarchical clustering of these eight DEPs showed that normal and pneumonia samples were clustered into one group and KD samples were clustered into another group. This result indicated that the expression of these eight DEPs, especially PGLYRP1 and PECAM1, could be used to distinguish KD from both normal control and pneumonia.

## Discussion

Due to lack of understanding of the mechanism of KD and misdiagnosis or delayed diagnosis of KD, both the mortality of KD and complications of KD are increased 11, 12. To develop accurate diagnostic biomarkers that can distinguish KD from normal control and other similar febrile diseases such as pneumonia, we performed a urine proteomic analysis in patients with KD, pneumonia (two patients) and normal controls.

Our study identified a total of 43 DEPs in KD compared to normal control. Among them, 13 DEPs were differentially expressed in both KD and pneumonia. Vasopressin‐regulated water reabsorption was a common enriched pathway in both KD and pneumonia (two patients). Both KD and pneumonia are febrile diseases, and we speculated that these 13 DEPs and vasopressin‐regulated water reabsorption may be closely associated with febrile diseases.

A total of 30 particular DEPs in patients with KD which did not belong to the DEPs in pneumonia were identified. These 30 DEPs may not only play key roles in the pathogenesis of KD but also served as potential diagnostic biomarkers of KD. Moreover, the expression of eight of these 30 DEPs (PECAM1, PGLYRP1, AHCY, BHMT, EML3, ICOSLG, IGHV3‐23 and RTN4RL2) clustered normal and pneumonia samples into one group and clustered KD samples into another group, which heightens the importance of these eight DEPs in KD.

One of these eight DEPs was platelet endothelial cell adhesion molecule 1 (PECAM‐1), which was found for the first time to be down‐regulated in patients with KD at protein level compared to normal control and pneumonia (two patients) in our study. PECAM‐1 is a member of the immunoglobulin superfamily, which are major transmembrane glycoproteins mainly expressed on the surface of blood and vascular cells 13. As an adhesion molecule, PECAM‐1 was reported to play key roles in vascular biology such as maintaining the vascular permeability barrier, regulating the migration of monocytes and neutrophils through venular walls, and response of endothelial cell to fluid shear stress, angiogenesis, platelet function and thrombosis 1, 14. Previous studies have demonstrated that several polymorphisms of PECAM‐1 were involved with the development of coronary artery lesions of patients with KD 1, 2. Moreover, PECAM‐1 +373 A/G gene polymorphism was found to be associated with the presence of KD 2. We speculate that PECAM‐1 may play a crucial role in KD and may serve as a potential diagnostic biomarker and therapeutic target of KD. Moreover, PECAM‐1 may be used to distinguish KD from other febrile diseases. Further research is needed to explore the precise role of PECAM‐1 in KD.

Peptidoglycan recognition protein 1 (PGLYRP1) is another of these eight DEPs and was found for the first time to be up‐regulated in patients with KD compared to normal control and pneumonia in our study. It is a member of the PGLYRP family, which regulate innate immunity. No previous study has reported the association between PGLYRP1 and KD. The innate immune system was reported to play a crucial role in the processes of KD, due to elevated pathogen‐associated molecular patterns and damage‐associated molecular patterns in KD acute phase sera 15. In addition, treatment with innate immune Nod1 ligand could result in KD‐like coronary arteritis in mice 15. It can be speculated that PGLYRP1 is another biomarker used to distinguish KD from normal control and patients with pneumonia.

Immunoglobulin heavy variable 3‐23 (IGHV3‐23) was also one of the eight DEPs that was up‐regulated in KD compared to normal control. A previous study indicated that it is involved with antibody responses of the immune system 16. Abnormal immune responses to infectious agents have been indicated to play crucial roles in initiation of KD 17. IGHV3‐23 may play a role in KD by regulating immune responses.

Based on KEGG enrichment analysis, the renin–angiotensin system was a significantly enriched pathway in patients with KD that was not enriched in patients with pneumonia. No previous study has reported the association between the renin–angiotensin system and KD. However, activated renin–angiotensin system was reported to be closely associated with endothelial dysfunction and vascular oxidative stress in autoimmune and inflammatory disease models 18. Moreover, endothelial dysfunction has been found in patients with KD and CAAs. Increased oxidative stress was also found in patients with KD 19 and speculated to play a key role in KD by promoting vascular dysfunction and CAAs with increased production of reactive oxygen species 19. Hence, we speculate that the renin–angiotensin system may be involved with the pathogenesis of KD by regulating the functions of vascular endothelium. As an enriched protein of the renin–angiotensin system, MME (neprilysin) was down‐regulated in patients with KD while not differentially expressed in patients with pneumonia compared to normal control. MME is a type II transmembrane glycoprotein and a common acute lymphocytic leukemia antigen. It may be a potential regulator of KD by regulating the renin–angiotensin system.

According to the KD‐specific network, SHMT1 is a hub protein that was down‐regulated in patients with KD compared to normal control. SHMT1 is a pyridoxal phosphate‐containing enzyme that catalyzes the reversible conversion of serine and tetrahydrofolate to glycine and 5,10‐methylene tetrahydrofolate 20. The biological function of SHMT1 in KD needs further research. Six proteins of the KD‐specific PPI network (MME, BHMT, AHCY, RAB11B, PDZK1 and DPEP1) were solely differentially expressed in KD compared to normal control. Their interactions may play special roles in the pathogenesis of KD.

## Conclusions

In conclusion, our study provides new clues for understanding the pathogenesis of and developing novel diagnostic and therapeutic strategies for KD based on the common and specific DEPs of KD and pneumonia (two patients). Moreover, we identified several key DEPs, especially PECAM‐1 and PGLYRP1, that may play roles in KD and may serve as more accurate and sensitive biomarkers of KD to distinguish patients with KD from both normal controls and patients with other febrile diseases.

## Author contributions

ZD and HH conceived and designed the project. HD acquired the data. JC and DF analyzed and interpreted the data. ZD and HH wrote the paper.

## Conflict of interest

The authors declare no conflict of interest.

## Supporting information


**Fig. S1.** The top 15 most significantly enriched GO terms enriched in DEPs in pneumonia. The *x*‐axis represents the −log (*P*‐value) and the *y*‐axis represents the GO terms. (A) Biological process (BP); (B) Molecular function (MF); (C) Cellular component (CC).

**Table S1.** The differentially expressed proteins in pneumonia compared to normal control.
