# Supplementary material for: New biomarkers of Kawasaki disease identified by urine proteomic analysis
Source: FEBS Open Bio. 2018 Dec 20;9(2):265–75. doi: 10.1002/2211-5463.12563 (PMC6356163; doi:10.1002/2211-5463.12563)

**Supplementary Figure 1 The top 15 most significantly enriched GO terms enriched in DEPs in pneumonia.**

The x-axis represents the -log (*p*-value) and y-axis represents the GO terms.

A). Biological process (BP); B). Molecular function (MF); C). Cellular component (CC).


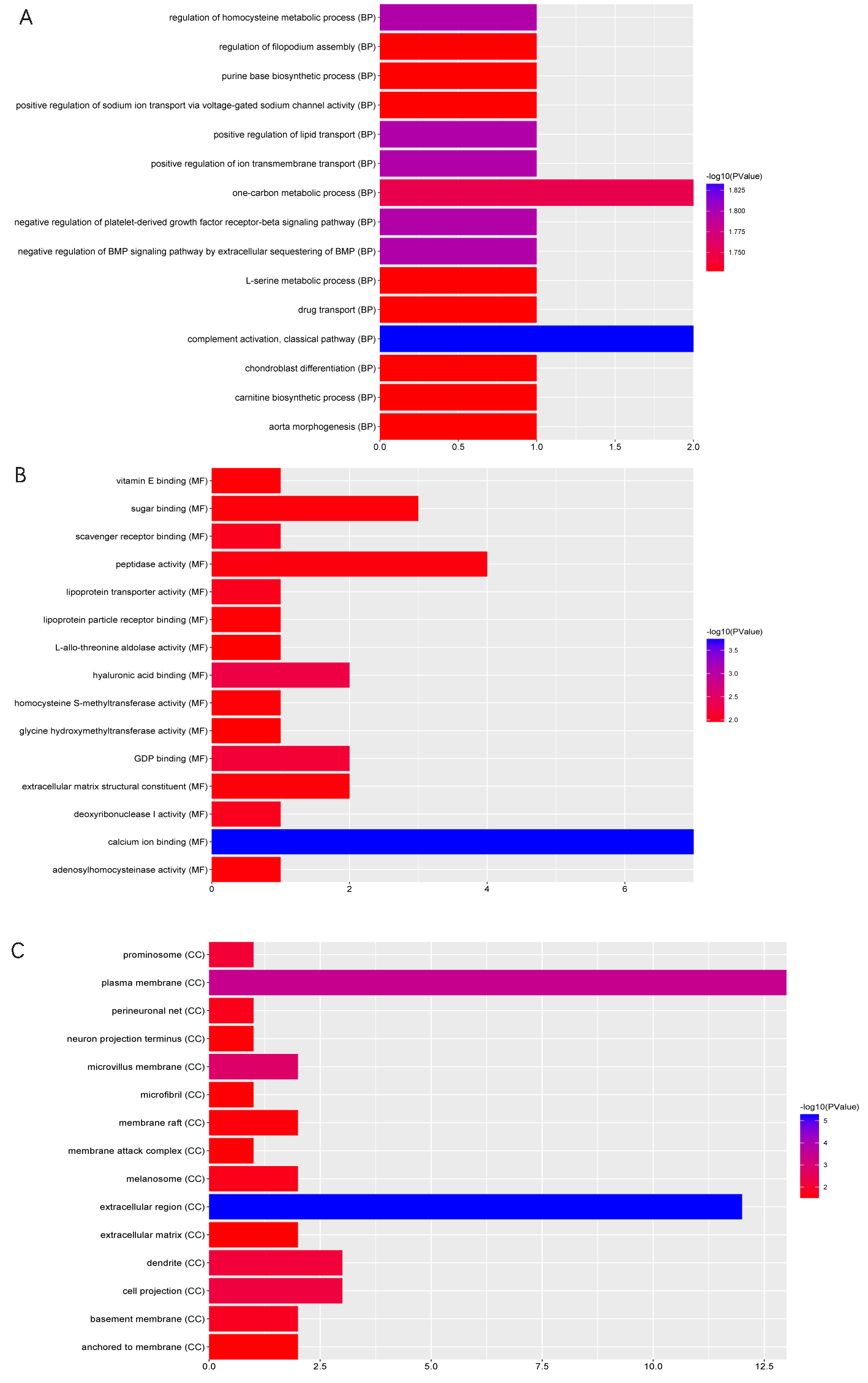

Supplement: Supplementary file 1 — Fig. S1. The top 15 most significantly enriched GO terms enriched in DEPs in pneumonia. The x‐axis represents the −log (P‐value) and the y‐axis represents the GO terms. (A) Biological process (BP); (B) Molecular function (MF); (C) Cellular component (CC). [file FEB4-9-265-s001.doc]
