# Supplementary material for: New biomarkers of Kawasaki disease identified by urine proteomic analysis
Source: FEBS Open Bio. 2018 Dec 20;9(2):265–75. doi: 10.1002/2211-5463.12563 (PMC6356163; doi:10.1002/2211-5463.12563)
Supplement: Supplementary file 2 — Table S1. The differentially expressed proteins in pneumonia compared to normal control. [file FEB4-9-265-s002.doc]

Table S1 The differentially expressed proteins in pneumonia compared to normal control

| **Accession** | **Description** | **Abundance Ratio** | **P.value** |
| --- | --- | --- | --- |
| P35052 | Glypican-1 OS=Homo sapiens GN=GPC1 PE=1 SV=2 | -1.946 | 0.000 |
| Q06830 | Peroxiredoxin-1 OS=Homo sapiens GN=PRDX1 PE=1 SV=1 | 2.309 | 0.001 |
| Q8IX04 | Ubiquitin-conjugating enzyme E2 variant 3 OS=Homo sapiens GN=UEVLD PE=1 SV=2 | -2.381 | 0.004 |
| P04083 | Annexin A1 OS=Homo sapiens GN=ANXA1 PE=1 SV=2 | 5.414 | 0.004 |
| P18510-3 | Isoform 3 of Interleukin-1 receptor antagonist protein OS=Homo sapiens GN=IL1RN | 4.360 | 0.004 |
| P68871 | Hemoglobin subunit beta OS=Homo sapiens GN=HBB PE=1 SV=2 | 2.452 | 0.006 |
| P26038 | Moesin OS=Homo sapiens GN=MSN PE=1 SV=3 | -2.151 | 0.007 |
| P36952 | Serpin B5 OS=Homo sapiens GN=SERPINB5 PE=1 SV=2 | 9.019 | 0.008 |
| P04004 | Vitronectin OS=Homo sapiens GN=VTN PE=1 SV=1 | -1.634 | 0.009 |
| Q5JWF2 | Guanine nucleotide-binding protein G(s) subunit alpha isoforms XLas OS=Homo sapiens GN=GNAS PE=1 SV=2 | -1.934 | 0.009 |
| P11597 | Cholesteryl ester transfer protein OS=Homo sapiens GN=CETP PE=1 SV=2 | -1.560 | 0.009 |
| P23468 | Receptor-type tyrosine-protein phosphatase delta OS=Homo sapiens GN=PTPRD PE=1 SV=2 | -2.033 | 0.009 |
| Q96PX8 | SLIT and NTRK-like protein 1 OS=Homo sapiens GN=SLITRK1 PE=1 SV=2 | -2.506 | 0.010 |
| Q6UWR7 | Ectonucleotide pyrophosphatase/phosphodiesterase family member 6 OS=Homo sapiens GN=ENPP6 PE=1 SV=2 | -1.887 | 0.010 |
| O43895 | Xaa-Pro aminopeptidase 2 OS=Homo sapiens GN=XPNPEP2 PE=2 SV=3 | -1.988 | 0.010 |
| Q14982-4 | Isoform 4 of Opioid-binding protein/cell adhesion molecule OS=Homo sapiens GN=OPCML | -2.070 | 0.010 |
| P04406 | Glyceraldehyde-3-phosphate dehydrogenase OS=Homo sapiens GN=GAPDH PE=1 SV=3 | 1.657 | 0.011 |
| P01857 | Ig gamma-1 chain C region OS=Homo sapiens GN=IGHG1 PE=1 SV=1 | 2.789 | 0.012 |
| P23528 | Cofilin-1 OS=Homo sapiens GN=CFL1 PE=1 SV=3 | 1.945 | 0.012 |
| P34896 | Serine hydroxymethyltransferase, cytosolic OS=Homo sapiens GN=SHMT1 PE=1 SV=1 | -2.353 | 0.012 |
| O14594 | Neurocan core protein OS=Homo sapiens GN=NCAN PE=1 SV=3 | -2.762 | 0.012 |
| Q9NRX4 | 14 kDa phosphohistidine phosphatase OS=Homo sapiens GN=PHPT1 PE=1 SV=1 | -2.088 | 0.012 |
| Q6UY11 | Protein delta homolog 2 OS=Homo sapiens GN=DLK2 PE=2 SV=1 | -5.263 | 0.013 |
| Q08380 | Galectin-3-binding protein OS=Homo sapiens GN=LGALS3BP PE=1 SV=1 | -2.062 | 0.013 |
| Q92896-2 | Isoform 2 of Golgi apparatus protein 1 OS=Homo sapiens GN=GLG1 | -1.742 | 0.014 |
| P01610 | Ig kappa chain V-I region WEA OS=Homo sapiens PE=1 SV=1 | 2.572 | 0.014 |
| P24855 | Deoxyribonuclease-1 OS=Homo sapiens GN=DNASE1 PE=1 SV=1 | -1.656 | 0.016 |
| Q9P2E9 | Ribosome-binding protein 1 OS=Homo sapiens GN=RRBP1 PE=1 SV=4 | 2.204 | 0.016 |
| O95445 | Apolipoprotein M OS=Homo sapiens GN=APOM PE=1 SV=2 | 1.797 | 0.017 |
| P22392 | Nucleoside diphosphate kinase B OS=Homo sapiens GN=NME2 PE=1 SV=1 | 2.715 | 0.017 |
| Q96HD9 | N-acyl-aromatic-L-amino acid amidohydrolase (carboxylate-forming) OS=Homo sapiens GN=ACY3 PE=1 SV=1 | -2.033 | 0.017 |
| P35555 | Fibrillin-1 OS=Homo sapiens GN=FBN1 PE=1 SV=3 | -4.484 | 0.018 |
| Q96I82 | Kazal-type serine protease inhibitor domain-containing protein 1 OS=Homo sapiens GN=KAZALD1 PE=1 SV=1 | -1.524 | 0.018 |
| Q9UBC9 | Small proline-rich protein 3 OS=Homo sapiens GN=SPRR3 PE=1 SV=2 | 2.836 | 0.019 |
| Q8NC54 | Keratinocyte-associated transmembrane protein 2 OS=Homo sapiens GN=KCT2 PE=2 SV=2 | -1.757 | 0.019 |
| P51149 | Ras-related protein Rab-7a OS=Homo sapiens GN=RAB7A PE=1 SV=1 | 2.578 | 0.021 |
| P69905 | Hemoglobin subunit alpha OS=Homo sapiens GN=HBA1 PE=1 SV=2 | 2.609 | 0.023 |
| Q6DKI7 | Transmembrane protein PVRIG OS=Homo sapiens GN=PVRIG PE=2 SV=1 | -3.040 | 0.023 |
| Q8WW52 | Protein FAM151A OS=Homo sapiens GN=FAM151A PE=2 SV=2 | -1.934 | 0.024 |
| P21266 | Glutathione S-transferase Mu 3 OS=Homo sapiens GN=GSTM3 PE=1 SV=3 | -1.812 | 0.026 |
| Q5T2W1 | Na(+)/H(+) exchange regulatory cofactor NHE-RF3 OS=Homo sapiens GN=PDZK1 PE=1 SV=2 | -2.469 | 0.026 |
| Q9UBG3 | Cornulin OS=Homo sapiens GN=CRNN PE=1 SV=1 | 4.490 | 0.027 |
| A6NGN9 | IgLON family member 5 OS=Homo sapiens GN=IGLON5 PE=2 SV=4 | -1.818 | 0.028 |
| P20339 | Ras-related protein Rab-5A OS=Homo sapiens GN=RAB5A PE=1 SV=2 | -3.333 | 0.029 |
| P01834 | Ig kappa chain C region OS=Homo sapiens GN=IGKC PE=1 SV=1 | 2.661 | 0.030 |
| P20916 | Myelin-associated glycoprotein OS=Homo sapiens GN=MAG PE=1 SV=1 | -1.704 | 0.030 |
| Q16706 | Alpha-mannosidase 2 OS=Homo sapiens GN=MAN2A1 PE=1 SV=2 | -2.604 | 0.032 |
| P0DMV9 | Heat shock 70 kDa protein 1B OS=Homo sapiens GN=HSPA1B PE=1 SV=1 | 1.933 | 0.032 |
| P48637 | Glutathione synthetase OS=Homo sapiens GN=GSS PE=1 SV=1 | 2.130 | 0.033 |
| P62070-4 | Isoform 4 of Ras-related protein R-Ras2 OS=Homo sapiens GN=RRAS2 | -2.433 | 0.038 |
| P06396 | Gelsolin OS=Homo sapiens GN=GSN PE=1 SV=1 | 1.511 | 0.038 |
| Q9UGT4 | Sushi domain-containing protein 2 OS=Homo sapiens GN=SUSD2 PE=1 SV=1 | -1.845 | 0.039 |
| Q9NR99 | Matrix-remodeling-associated protein 5 OS=Homo sapiens GN=MXRA5 PE=2 SV=3 | -2.208 | 0.040 |
| Q9NY72 | Sodium channel subunit beta-3 OS=Homo sapiens GN=SCN3B PE=1 SV=1 | -3.717 | 0.041 |
| Q07954 | Prolow-density lipoprotein receptor-related protein 1 OS=Homo sapiens GN=LRP1 PE=1 SV=2 | -3.058 | 0.042 |
| P62979 | Ubiquitin-40S ribosomal protein S27a OS=Homo sapiens GN=RPS27A PE=1 SV=2 | -1.808 | 0.042 |
| Q9Y2S2 | Lambda-crystallin homolog OS=Homo sapiens GN=CRYL1 PE=1 SV=3 | -1.942 | 0.048 |
| Q99497 | Protein deglycase DJ-1 OS=Homo sapiens GN=PARK7 PE=1 SV=2 | 2.214 | 0.048 |
| Q16849 | Receptor-type tyrosine-protein phosphatase-like N OS=Homo sapiens GN=PTPRN PE=1 SV=1 | -3.876 | 0.048 |
| P37802-2 | Isoform 2 of Transgelin-2 OS=Homo sapiens GN=TAGLN2 | 3.657 | 0.049 |
| B9A064 | Immunoglobulin lambda-like polypeptide 5 OS=Homo sapiens GN=IGLL5 PE=2 SV=2 | 2.956 | 0.049 |
| P29373 | Cellular retinoic acid-binding protein 2 OS=Homo sapiens GN=CRABP2 PE=1 SV=2 | 4.500 | 0.050 |

Abundance Ratio, the abundance ratio (pneumonia / normal control).
